# Effects of Remote, Virtual, or Hybrid Cardiac Rehabilitation Supported by mHealth in Patients With Heart Failure: Systematic Review and Meta-Analysis

**DOI:** 10.2196/90422

**Published:** 2026-07-21

**Authors:** Kaidong Shao, Chunqiu Liu, Tianshu Li, Huiyan Qu, Hua Zhou

**Affiliations:** 1 Institute of Cardiovascular Disease of Integrated Traditional Chinese and Western Medicine, Shuguang Hospital Affiliated to Shanghai University of Traditional Chinese Medicine, No. 528, Zhangheng Road, Pudong New Area, Shanghai, China, 86 13258672918; 2 Department of Cardiovascular Medicine, Shuguang Anhui Hospital Affiliated to Shanghai University of Traditional Chinese Medicine, Hefei, Anhui, China

**Keywords:** mobile health, mHealth, heart failure, meta-analysis, center-based cardiac rehabilitation, remote cardiac rehabilitation, virtual cardiac rehabilitation, hybrid cardiac rehabilitation

## Abstract

**Background:**

Structured exercise is a key component of cardiac rehabilitation (CR) for patients with heart failure (HF), but access to center-based cardiac rehabilitation (CBCR) is often limited. Mobile health (mHealth) platforms enable remote, virtual, or hybrid cardiac rehabilitation (RVH-CR) delivery.

**Objective:**

This study aimed to evaluate the effectiveness and safety of structured, exercise-focused RVH-CR supported by mHealth compared with usual care or CBCR in patients with heart failure with reduced ejection fraction (HFrEF) or in HF populations predominantly comprising patients with HFrEF.

**Methods:**

We searched PubMed, Web of Science, MEDLINE via Ovid, Cochrane CENTRAL, and CINAHL Complete from inception to April 27, 2026. Randomized controlled trials comparing mHealth-supported RVH-CR with usual care or CBCR were included. The primary outcome was exercise capacity, assessed by peak oxygen uptake (VO_2_ peak) and 6-minute walk distance (6MWD). Secondary outcomes included health-related quality of life and safety. Data were pooled using random-effects meta-analysis stratified by comparator. Risk of bias was assessed with the Cochrane Risk of Bias Tool version 2, and evidence certainty was evaluated using GRADE (Grading of Recommendations Assessment, Development, and Evaluation).

**Results:**

Eight randomized controlled trials with 1368 patients were included. In the CBCR comparison, mHealth-supported RVH-CR showed a statistically significant greater improvement in VO_2_ peak than CBCR (mean difference [MD] 0.82, 95% CI 0.06-1.57; *P*=.03), although this finding was based on a limited number of trials. Compared with usual care, mHealth-supported RVH-CR was associated with improved 6MWD (MD 22.99, 95% CI 1.15-44.82; *P*=.04). Single-trial estimates suggested improvements in VO_2_ peak (MD 2.50, 95% CI 0.88-4.12) and Minnesota Living with Heart Failure Questionnaire scores (standardized MD −0.57, 95% CI −0.98 to −0.17; *P*<.01) versus usual care. The certainty of evidence ranged from low to moderate. No intervention-related deaths or serious adverse events were reported, but sparse events and short follow-up limited conclusions regarding safety.

**Conclusions:**

The effects of structured RVH-CR supported by mHealth differed according to comparator type, but the certainty of evidence ranged from low to moderate. Compared with usual care, mHealth-supported RVH-CR was associated with improved 6MWD. Compared with CBCR, mHealth-supported RVH-CR showed a significantly greater improvement in VO_2_ peak in a limited number of trials, but superiority, equivalence, or noninferiority to CBCR cannot be concluded. Because usual care and CBCR are clinically distinct comparators, no single overall effect across comparator types should be inferred. Future studies should assess long-term outcomes and standardize structured exercise protocols across RVH-CR models.

## Introduction

### Background

Heart failure (HF) is a complex clinical syndrome in which structural or functional cardiac abnormalities impair ventricular filling or ejection, giving rise to characteristic symptoms and signs [1]. HF remains a significant public health challenge globally [2,3]. Guidelines emphasize comprehensive disease management for patients with HF, including evidence-based pharmacotherapy, cardiac rehabilitation (CR), and structured follow-up [4,5]. Among HF phenotypes, heart failure with reduced ejection fraction (HFrEF) is clinically distinct and has been widely studied [6]. Multiple American Heart Association clinical practice guidelines endorse CR as an effective strategy for reducing mortality and improving quality of life across the spectrum of cardiovascular disease [7-9]. Core components of CR programs include patient assessment, individualized exercise training, nutritional counseling, psychological support, and health education, delivered by multidisciplinary teams comprising physicians, nurses, exercise physiologists, behavioral health specialists, dietitians, physical therapists, and respiratory therapists [10].

Exercise-based CR has been shown to improve functional capacity and exercise tolerance, reduce overall and HF-specific hospitalization rates, and enhance health-related quality of life (HRQoL) [11]. Despite these benefits, referral and participation rates for center-based cardiac rehabilitation (CBCR) are low globally, limited by transportation, social support, and socioeconomic factors [12,13].

To address these access barriers, CR delivery has expanded beyond traditional facility-based programs to include remote, virtual, and hybrid models that may be delivered in the home, community centers, fitness facilities, or other noncenter settings [9,14,15]. Contemporary consensus documents emphasize delivery mode rather than location: virtual CR refers to synchronous, real-time audiovisual delivery; remote CR refers to asynchronous delivery with clinician monitoring, feedback, or communication; and hybrid CR combines facility-based, virtual, and/or remote components [16,17]. Digital technologies are increasingly integrated into CR delivery, offering solutions to the logistical challenges inherent to CBCR while expanding the reach of structured rehabilitation care [10,17].

In recent years, the proliferation of smartphones and mobile apps has driven growing interest in mobile health (mHealth)-supported remote, virtual, and hybrid cardiac rehabilitation (RVH-CR) delivery [18]. mHealth refers to the use of mobile and wireless technologies to support the achievement of health objectives, encompassing smartphones, tablets, mobile apps, SMS, wearable activity trackers, biosensors, and connected digital platforms that enable bidirectional communication and real-time monitoring of health data between patients and clinicians [19,20].

### Objectives

In recent clinical trials, mHealth-supported RVH-CR has been evaluated for its impact on patients with HF. However, there remains a lack of systematic reviews focusing specifically on exercise-based, structured RVH-CR supported by mHealth in patients with HFrEF or HFrEF-predominant HF populations. A meta-analysis conducted by Li et al [21] in 2025 compared the effects of mHealth home-based CR interventions versus usual care and CBCR across different cardiovascular conditions but lacked a specific focus on HF. Furthermore, the majority of systematic reviews [22-25] have focused on the impact of remote technologies on HF prognosis, with limited attention to exercise-based telerehabilitation modalities (such as videoconferencing platforms, computer-based systems, smartphone applications, or web-based home monitoring systems).

Throughout this review, we use “mHealth-supported RVH-CR” as the umbrella term for eligible interventions. “Remote,” “virtual,” and “hybrid” refer to CR delivery models, whereas “mHealth” refers to the portable, wireless, or connected digital technology layer supporting exercise prescription, monitoring, data transmission, clinician feedback, or supervision. “Home-based” is used only to describe the physical setting in which some intervention sessions occurred. “Telerehabilitation” is retained only when referring to terminology used by individual trials or previous reviews; otherwise, interventions are described using the standardized categories of RVH-CR. Given the emerging but still distinct literature on heart failure with preserved ejection fraction (HFpEF), the target population of this review was restricted to HFrEF or HFrEF-predominant HF populations to maintain clinical and pathophysiological coherence.

This systematic review aimed to identify and synthesize evidence from randomized clinical trials comparing the efficacy of mHealth-supported RVH-CR versus usual care or supervised CBCR in patients with HFrEF or HFrEF-predominant HF populations.

## Methods

This systematic review and meta-analysis were developed and reported according to the PRISMA (Preferred Reporting Items for Systematic Reviews and Meta-Analyses) guidelines (Checklist 1) [26]. The protocol was registered in PROSPERO (CRD420251162078).

### Search Strategy

We searched 5 databases (PubMed, Web of Science, MEDLINE via the Ovid interface, Cochrane CENTRAL, and CINAHL Complete) from their inception to April 27, 2026. We also manually checked the reference lists of related studies and reviews. The search strategy combined MeSH terms and keywords adjusted for each database. The electronic search strategy was developed by HZ after discussions with the other authors (Multimedia Appendix 1). Key search terms covered the following 4 concepts: “heart failure,” “cardiac rehabilitation,” “mobile applications,” and “exercise.”

### Selection Criteria

Studies were eligible if they met all of the following criteria: (1) the participants were adults (aged ≥18 y) diagnosed with HFrEF, or individuals with HF predominantly characterized by HFrEF. Trials were considered HFrEF-predominant if eligibility criteria specified reduced left ventricular ejection fraction, or if the enrolled population with HF was predominantly composed of patients with reduced left ventricular ejection fraction, as reported by the trial authors; (2) interventions were not classified as mHealth-supported RVH-CR solely because they used videoconferencing, telephone support, SMS, or general telehealth communication. To be eligible, the intervention had to combine structured exercise-based CR with a patient-facing portable, wireless, or connected digital technology that supported exercise prescription, execution, physiologic or activity monitoring, patient-generated data transmission, clinician feedback, or supervision; (3) the control group consisted of either usual care (routine outpatient follow-up and general lifestyle advice without structured exercise or mHealth technology) or CBCR (supervised, facility-based exercise training and face-to-face follow-up); (4) the study design was a randomized controlled trial (RCT); and (5) at least one prespecified outcome was reported—exercise capacity (peak oxygen uptake [VO_2_ peak] or 6-minute walk distance [6MWD]) or HRQoL.

Studies were excluded if (1) SMS, telephone, or videoconferencing was used solely for one-way reminders, general health education, or administrative communication, without structured exercise management, patient-generated data capture, or rehabilitation-specific feedback; (2) the full text was unavailable or the study was incomplete; or (3) the publication was not an original report (eg, review, conference abstract, or commentary).

### Study Selection

All database search results were imported into EndNote X9 (Clarivate) for automated deduplication, followed by manual verification to ensure accuracy. Two reviewers (KS and CL) independently screened titles and abstracts against the eligibility criteria, followed by a full-text review of potentially eligible records. Discrepancies at any stage were resolved through discussion, and unresolved disagreements were adjudicated by a third reviewer (HZ).

### Data Extraction

Two reviewers (KS and CL) independently extracted data using a standardized form developed in Microsoft Excel. Extracted items included study characteristics, participant demographics, intervention and control group details, and outcome measures. Discrepancies were resolved by consensus, with HZ serving as an arbiter when necessary. When data were missing or unclear, the corresponding authors were contacted by email to request the required information.

### Risk of Bias and Quality of Evidence

Two reviewers (KS and TL) independently assessed the risk of bias (RoB) of each included RCT using the Cochrane Risk of Bias Tool version 2 (RoB 2), which evaluates 5 domains: the randomization process, deviations from intended interventions, missing outcome data, measurement of the outcome, and selection of the reported result. Each domain was rated as low risk, some concerns, or high risk, with an overall judgment derived according to RoB 2 guidance. Disagreements were resolved through discussion, with author HQ adjudicating unresolved cases.

The certainty of evidence for each outcome was independently assessed by 2 reviewers (KS and TL) using the GRADE (Grading of Recommendations Assessment, Development, and Evaluation) framework through GRADEpro software (Evidence Prime). Evidence certainty was rated across 5 domains—RoB, inconsistency, indirectness, imprecision, and publication bias—and classified as high, moderate, low, or very low.

### Statistical Analysis

The primary outcome was exercise capacity, operationalized as VO_2_ peak and 6MWD. Secondary outcomes were HRQoL, assessed using the 36-Item Short Form Health Survey (SF-36) and the Minnesota Living with Heart Failure Questionnaire (MLHFQ). Where outcomes were amenable to quantitative synthesis, pooled effect estimates with 95% CIs were calculated using a random-effects model (DerSimonian-Laird method), which was prespecified to account for anticipated clinical and methodological heterogeneity across trials. Separate meta-analyses were conducted for 2 predefined comparisons: mHealth-supported RVH-CR versus usual care and mHealth-supported RVH-CR versus CBCR.

The meta-analyses were conducted using change-from-baseline values rather than postintervention values. For each outcome, pooled estimates represent between-group differences in change over the intervention period. As all outcomes were continuous, the mean difference (MD) was used when studies used identical measurement instruments, whereas the standardized mean difference (SMD) was calculated when different instruments or scale versions were used. Effect sizes were interpreted according to Cohen conventions: small (SMD 0.2), medium (SMD 0.5), and large (SMD 0.8). Statistical heterogeneity was quantified using the Cochran *Q* test and the *I*^2^ statistic. Substantial heterogeneity was predefined as *I*^2^>50%, *Q* test, *P*<.05, and clinically meaningful variability in the direction or magnitude of effects. Assessment of publication bias via funnel plot asymmetry and Egger regression test was not performed, as fewer than 10 studies were available for any single comparison—a threshold below which such tests lack adequate statistical power. All analyses were performed using Stata version 18.0 (StataCorp).

## Results

### Study Selection

**Figure 1.**
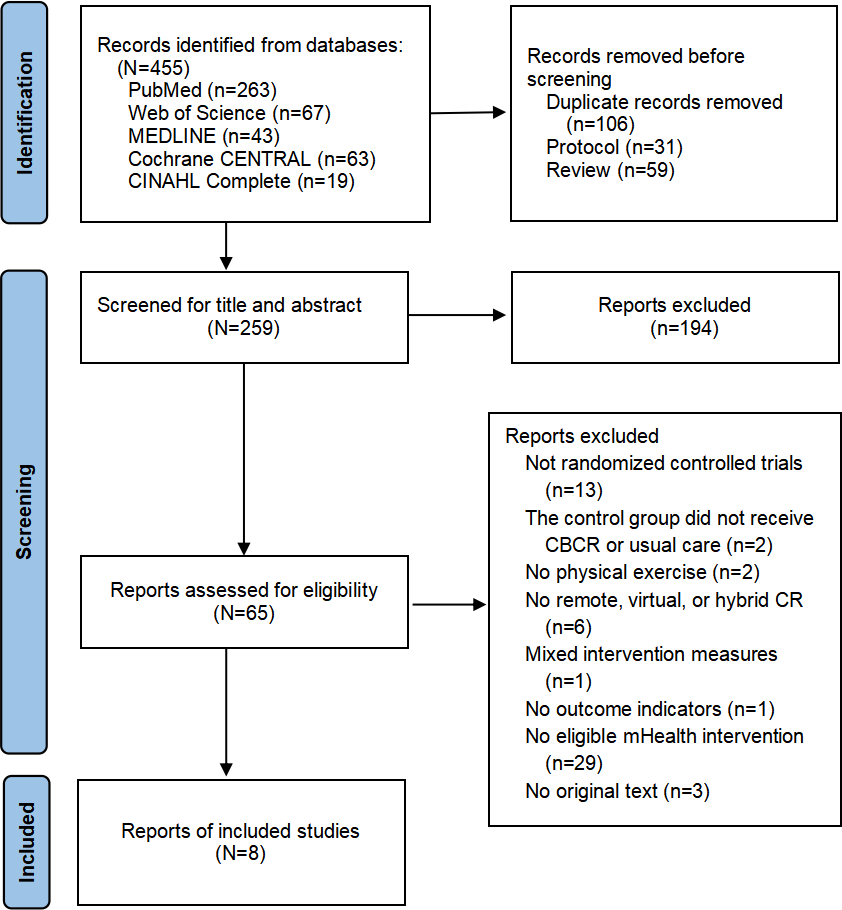
Study selection. CBCR: center-based cardiac rehabilitation; CR: cardiac rehabilitation; mHealth: mobile health.

As shown in Figure 1, 455 records were identified from 5 databases. After removing duplicates, protocols, and reviews, 259 records were screened by title and abstract. Of these, 194 were excluded, and 65 reports were assessed for eligibility.

After full-text assessment, 57 reports were excluded because they were not RCTs, did not include an eligible control group, did not include a physical exercise or mHealth intervention, did not evaluate RVH-CR delivered outside traditional center-based settings, used mixed interventions, lacked relevant outcomes, or had no original text. Finally, 8 studies were included in the systematic review.

### Study Characteristics

The 8 included studies [27-34] were published between 2010 and 2025. Five studies [30-34] were conducted in Europe, 2 [28,29] in Asia, and 1 [27] in Oceania. All studies were prospective, parallel-group RCTs that investigated mHealth-supported RVH-CR, with most patient exercise sessions conducted in the home setting. The included trials enrolled patients with HFrEF or HFrEF-predominant populations with HF. A condensed summary of the included studies is provided in Table 1, and detailed study characteristics are provided in Multimedia Appendix 2.

**Table 1.** Summary characteristics of the included randomized controlled trials.^a^

Study	Country	Total sample size, n (intervention:control)	Comparator	Eligibility rationale for mHealth-supported CR^b^ classification
Hwang et al [27], 2017	Australia	53 (24:29)	CBCR^c^ exercise plus education	Qualified as mHealth-supported^d^ virtual CR because real-time video conferencing was combined with structured exercise supervision and patient-facing home monitoring of blood pressure and oxygen saturation rather than used only for remote communication.
Schmidt et al [33], 2025	Portugal	120 (75:45)	24-session supervised CBCR	Qualified because smartwatch-based exercise data upload and asynchronous clinician review supported structured home exercise prescription, monitoring, and feedback.
Piotrowicz et al [30], 2010	Poland	152 randomized; 131 included in final analysis (75:56)	Supervised center-based cycle-ergometer CR	Qualified because portable ECG^e^ telemonitoring, mobile phone transmission, audiovisual prompts, and clinician contact were used to guide and supervise structured home exercise training.
Piotrowicz et al [31], 2021	Poland	850 randomized; QoL^f^ analysis included 768 (377:391)	Usual care or observation with lifestyle and self-management advice	Qualified because hybrid CR incorporated connected ECG, blood pressure, weight, and device-related monitoring through a monitoring center to support structured CR delivery.
Nagatomi et al [28], 2022	Japan	30 (15:15)	Usual pharmacological and nonpharmacological care	Qualified because Fitbit-based activity monitoring, symptom/vital self-monitoring, and app/telephone feedback generated patient data used to support individualized exercise and clinical feedback.
Peng et al [29], 2018	China	98 (49:49)	Discharge education plus routine outpatient follow-up	Eligible because digital communication was integrated with structured exercise management, monitoring, and feedback rather than used only for education or reminders.
Piotrowicz et al [32], 2015	Poland	111 randomized; 107 included in final analysis (75:32)	Lifestyle and self-management advice; no supervised exercise	Eligible because portable connected monitoring was used to supervise and adjust structured Nordic walking training.
Lundgren et al [34], 2023	Norway	61 (31:30)	Usual care; guideline-based exercise encouraged	Eligible because synchronous CR was used to deliver structured exercise with active clinician supervision; however, it represents the broadest boundary of the mHealth-supported category and was retained because delivery depended on patient-facing connected digital technology.

a Values are mean or mean (SD) unless otherwise indicated. Randomized and final analysis samples are both reported when available. Detailed study characteristics are provided in Multimedia Appendix 2.

b CR: cardiac rehabilitation.

c CBCR: center-based cardiac rehabilitation.

d mHealth: mobile health.

e ECG: electrocardiogram.

f QoL: quality of life.

mHealth platforms in the included studies used broadband-connected laptops, tablet computers with videoconferencing systems, smartphones, smartwatches, and EHO (Pro Plus Company) devices [27-34]. Physiological monitoring was conducted using automated blood pressure monitors [27], electrocardiogram monitoring and transmission systems [30,31], wearable devices or smartwatches [28,33], and heart rate monitoring in a subset of participants [34]. Intervention durations ranged from 8 to 12 weeks. Exercise frequencies varied from daily to 5 times per week. The main type of exercise was aerobic exercise. Three studies also provided resistance training [28,29,31]. Two studies offered strength training [27,31]. Exercise prescriptions were generally individualized according to baseline functional capacity, symptoms, heart rate response, Borg perceived exertion score, or remotely transmitted physiological data. In most trials, physical therapists or multidisciplinary rehabilitation teams monitored progress and adjusted exercise intensity according to patient condition and training performance [27-29,33,34]. Psychological support was provided in 3 studies [30-32], nutritional counseling in 1 study [28], and lifestyle modification or self-management education was included in several studies [27,29,33,34].

In 3 studies [27,30,33], the comparator was CBCR. In the remaining 5 studies [28,29,31,32,34], it was usual care. CBCR comprised supervised, facility-based exercise training and face-to-face educational or counseling sessions. Usual care consisted of routine outpatient follow-up, general recommendations for lifestyle modification, and self-management guidance, but excluded structured exercise training.

### RoB

**Figure 2.**
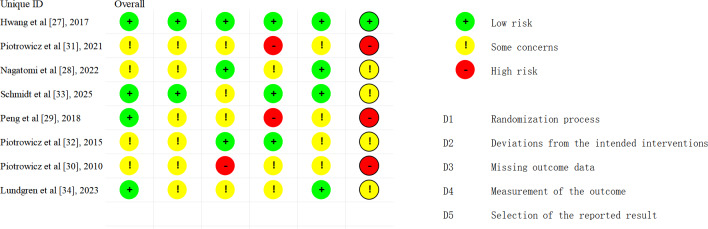
Each risk of bias domain for each randomized controlled trial [27-34].

The included studies were assessed for RoB. One study [27] was considered to be at low risk because of concealed allocation, blinded outcome assessment, intention-to-treat analysis, trial registration, and minimal missing outcome data. Four studies [28,32-34] were rated as having some concerns, mainly due to the open-label nature of the interventions, incomplete reporting of allocation concealment or assessor blinding, baseline imbalance, or incomplete primary outcome data. Two studies [29,31] were judged to be at high risk primarily because their key outcomes were self-reported quality-of-life measures assessed in unblinded participants, whereas 1 study [30] was at high risk due to differential attrition and reliance on complete-case analysis. The most frequent sources of bias were lack of participant blinding, subjective outcome assessment, missing outcome data, and insufficient information on prespecified analysis plans. Therefore, pooled estimates for quality-of-life outcomes should be interpreted with caution, while findings based on objective exercise capacity outcomes, such as VO_2_ peak and 6MWD, are likely to be less vulnerable to measurement bias but remain affected by attrition and incomplete blinding in selected trials. The RoB judgments are summarized in Figure 2. The specific details can be found in Multimedia Appendix 3.

### Outcome Measures

#### Exercise Capacity

Exercise capacity was assessed using VO_2_ peak and 6MWD. Because the control groups differed clinically across studies, results were interpreted separately for comparisons with CBCR and usual care. The overall pooled estimates combining CBCR and usual care were not used as the primary basis for interpretation, because CBCR was an active exercise-based intervention, whereas usual care generally did not include structured exercise training. Therefore, we did not interpret any overall pooled effect combining usual care and CBCR, and the results below are presented separately for each comparator.

**Figure 3.**
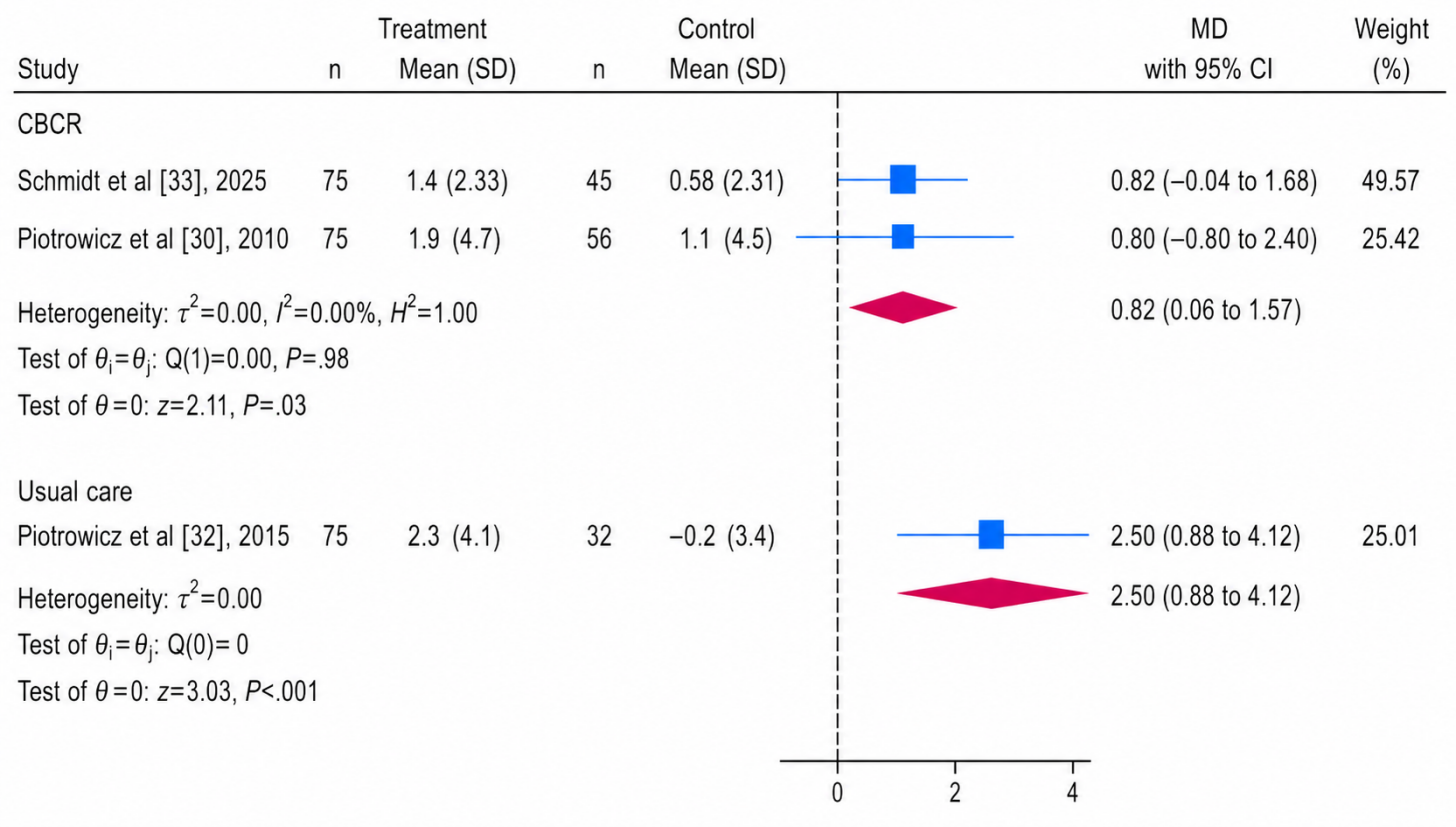
Forest plot showing the comparator-stratified effect of mobile health (mHealth)–supported RVH-CR on VO_2_ peak [30,32,33]. CBCR: center-based cardiac rehabilitation; MD: mean difference; RVH-CR: remote, virtual, or hybrid cardiac rehabilitation; VO_2_ peak: peak oxygen uptake.

VO_2_ peak was reported in 4 studies [30,32-34]. Three studies were included in the quantitative synthesis [30,32,33]. The study by Lundgren et al [34] was not included in the meta-analysis because postintervention VO_2_ peak values were not reported, which precluded the calculation of change-from-baseline values and corresponding effect estimates. This study was, therefore, summarized narratively. In the CBCR subgroup, mHealth-supported RVH-CR showed a statistically significant greater improvement in VO_2_ peak than CBCR (MD 0.82, 95% CI 0.06-1.57; *P*=.03; *I*^2^=0%; Figure 3). One trial comparing mHealth-supported RVH-CR with usual care also showed a greater improvement in VO_2_ peak in the intervention group (MD 2.50, 95% CI 0.88-4.12; Figure 3). Because this estimate was derived from a single trial, it should be interpreted as study-specific evidence rather than a pooled effect. It should not be considered evidence of established superiority over CBCR, particularly because the included trials were not designed to test superiority, equivalence, or noninferiority between delivery models.

**Figure 4.**
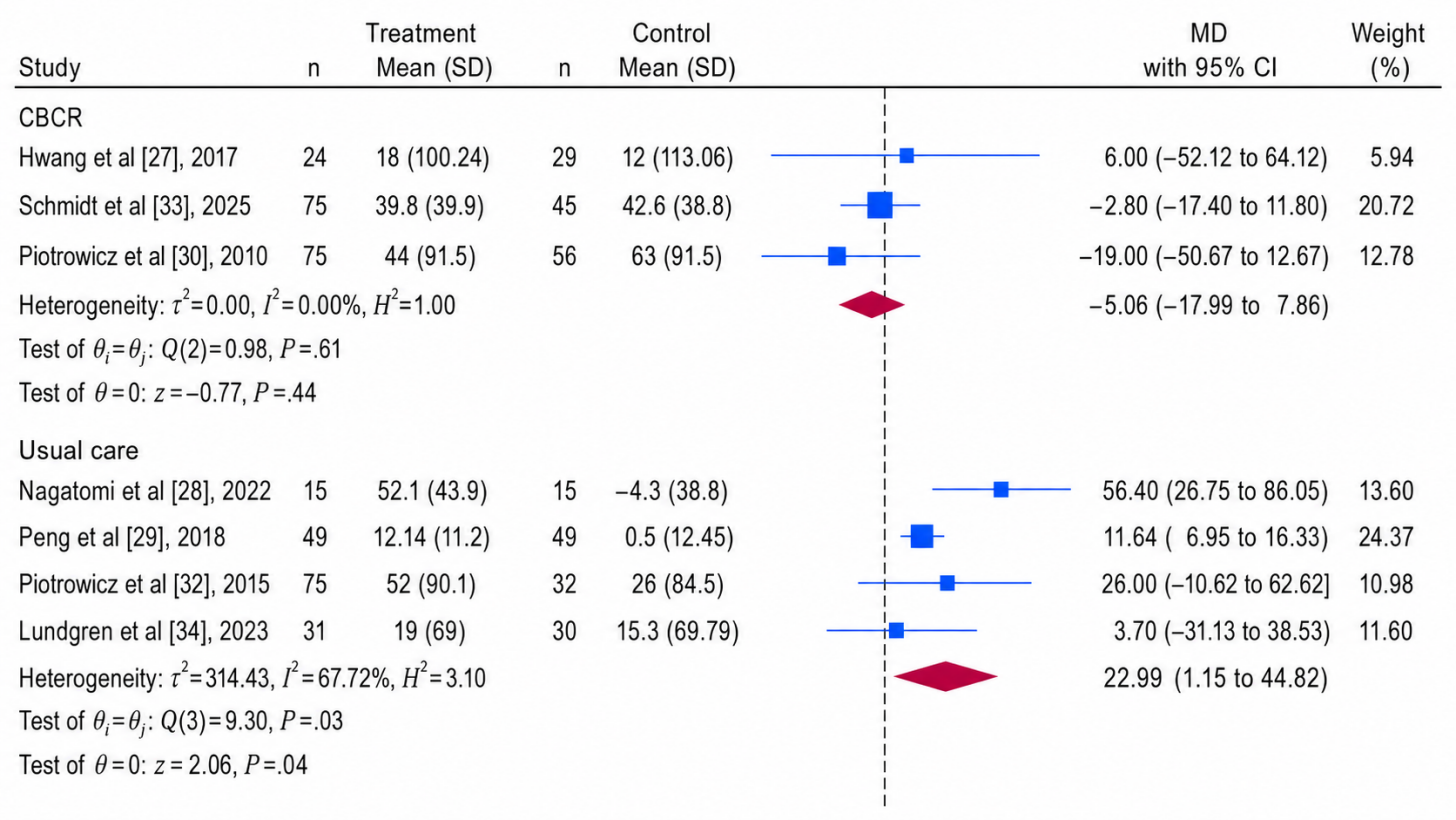
Forest plot showing the comparator-stratified effect of mobile health (mHealth)–supported RVH-CR on 6MWD [27-30,32-34]. 6MWD: 6-minute walk distance; CBCR: center-based cardiac rehabilitation; MD: mean difference; RVH-CR: remote, virtual, or hybrid cardiac rehabilitation.

Seven studies reported 6MWD and were included in the updated meta-analysis [27-30,32-34]. The results are shown in Figure 4. In the CBCR subgroup, mHealth-supported RVH-CR was not associated with a statistically significant difference in 6MWD compared with CBCR (MD −5.06, 95% CI −17.99 to 7.86; *P*=.44), with no observed heterogeneity across trials (*I*²=0%) [27,30,33]. In the usual care subgroup, mHealth-supported RVH-CR was associated with a significant improvement in 6MWD compared with usual care (MD 22.99, 95% CI 1.15-44.82; *P*=.04) [28,29,32,34]. However, heterogeneity in this subgroup was moderate to substantial (*I*²=67.72%), indicating meaningful between-study variability in effect estimates.

This heterogeneity indicates variability in treatment effects across trials and may reflect differences in patient populations, baseline functional capacity, intervention intensity, exercise frequency, remote monitoring strategies, adherence support, and the content of usual care. Therefore, the pooled 6MWD estimate should be interpreted as the average effect of a structured rehabilitation package supported by mHealth across heterogeneous intervention models, rather than as the isolated effect of mHealth technology itself.

#### HRQoL

**Figure 5.**
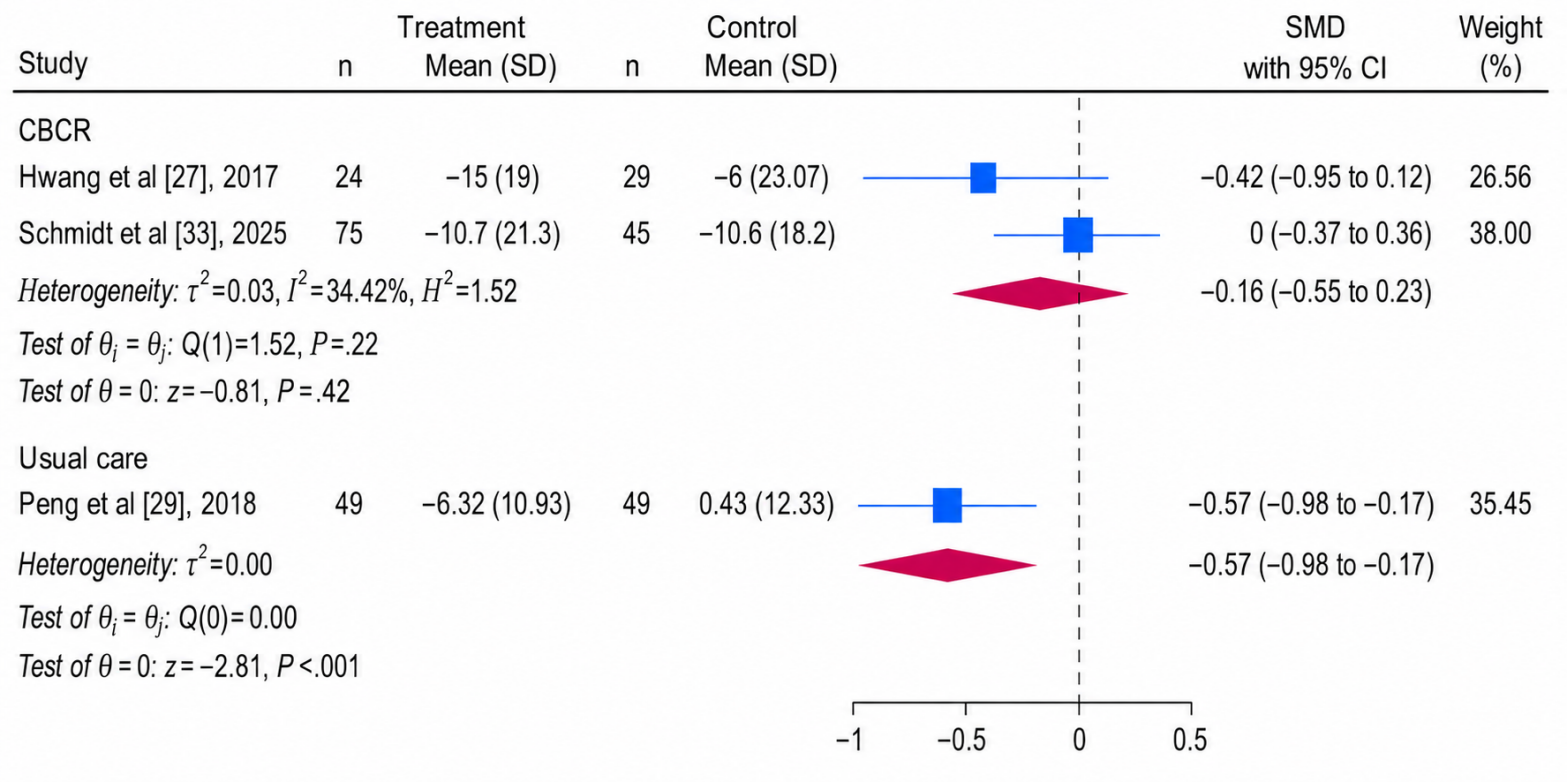
Forest plot showing the comparator-stratified effect of mobile health (mHealth)–supported RVH-CR on the Minnesota Living With Heart Failure Questionnaire [27,29,33]. CBCR: center-based cardiac rehabilitation; RVH-CR: remote, virtual, or hybrid cardiac rehabilitation; SMD: standardized mean difference.

HRQoL was assessed using the MLHFQ and SF-36 [27,29-33]. For all HRQoL analyses, effect directions were coded so that values favoring mHealth-supported RVH-CR indicated better HRQoL. Three studies assessed HRQoL using the MLHFQ [27,29,33]. Higher MLHFQ scores indicate worse HRQoL. The results are shown in Figure 5. In the CBCR subgroup, mHealth-supported RVH-CR showed no significant difference compared with CBCR (SMD −0.16, 95% CI −0.55 to 0.23; *P*=.42). One trial comparing mHealth-supported RVH-CR with usual care showed improved MLHFQ scores in the intervention group (SMD −0.57, 95% CI −0.98 to −0.17; *P*<.01). Because this usual care comparison was based on a single study, the result should be interpreted cautiously.

**Figure 6.**
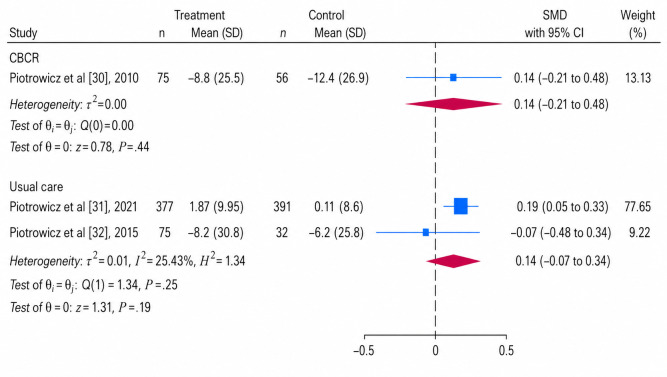
Forest plot showing the comparator-stratified effect of mobile health (mHealth)–supported RVH-CR on SF-36 [30-32]. CBCR: center-based cardiac rehabilitation; RVH-CR: remote, virtual, or hybrid cardiac rehabilitation; SF-36: 36-Item Short Form Health Survey; SMD: standardized mean difference.

Three studies assessed HRQoL using the SF-36 [30-32]. Higher SF-36 scores indicate better HRQoL. The results are shown in Figure 6. In the CBCR subgroup, 1 study found no significant difference between mHealth-supported RVH-CR and CBCR (SMD 0.14, 95% CI −0.21 to 0.48; *P*=.44) [30]. In the usual care subgroup, 2 studies showed no significant difference between mHealth-supported RVH-CR and usual care (SMD 0.14, 95% CI −0.07 to 0.34; *P*=.19) [31,32]. Therefore, the separated comparator analyses did not support a clear significant effect of mHealth-supported RVH-CR on SF-36 scores. These findings should be interpreted with caution because the SF-36 studies differed in sample size, intervention structure, and rehabilitation components.

#### Mortality, Hospitalization, and Adverse Events

Mortality, hospitalization, and major adverse cardiovascular events were not consistently defined or reported across the included studies, and these outcomes were not suitable for meta-analysis. Available safety data were, therefore, summarized narratively.

Across the 8 included studies, no report explicitly attributed death to the mHealth-supported RVH-CR intervention. Hwang et al [27] reported no deaths, cardiac arrest, syncope, or falls during exercise sessions. Nagatomi et al [28] reported no serious adverse events such as death or cardiac arrest; 3 readmissions occurred in each group and were not considered causally related to the intervention. Peng et al [29] reported no significant complications or adverse outcomes during the program. One study [30] reported no deaths or HF-related hospitalizations attributable to the rehabilitation program, although 1 patient in the standard rehabilitation group died following elective orthotopic heart transplantation. In the TELEREH-HF (Telerehabilitation in Heart Failure Patients) quality-of-life analysis, 2 deaths occurred in the hybrid telerehabilitation group and 2 in the usual care group during the 9-week intervention or observation period [31]. One study [32] reported no deaths or hospitalizations in either group and no cardiovascular implantable electronic device interventions during Nordic walking training. Schmidt et al [33] reported 1 death in the home-based group, which was not related to the exercise training program, and no hospitalizations, musculoskeletal injuries, or other exercise-related adverse events during the 12-week study period. Lundgren et al [34] reported no exercise session-related adverse events during real-time supervised home-based telerehabilitation; 1 death occurred in the control group before the first follow-up.

Overall, no deaths or serious adverse events were explicitly attributed to mHealth-supported RVH-CR in the included trials. However, these findings should be interpreted cautiously because follow-up periods were short, event numbers were small, and clinical end points were inconsistently defined and reported. Therefore, the available evidence is insufficient to establish definitive conclusions regarding all-cause mortality, cardiovascular mortality, hospitalization, major adverse cardiovascular events, or overall intervention safety.

### Certainty of Evidence

The certainty of evidence was assessed using the GRADE framework and is summarized in Table 2. Full domain-level judgments are provided in Multimedia Appendix 4. Because usual care and CBCR were clinically distinct comparators, certainty ratings were summarized separately by comparator type. Overall, the certainty was moderate for selected exercise capacity outcomes compared with CBCR and low for most usual-care and HRQoL comparisons. The most common reasons for downgrading were RoB, a small number of studies, imprecision, heterogeneity, and the subjective assessment of HRQoL outcomes.

**Table 2.** Summary of findings and certainty of evidence by outcome and comparator.

Outcome	Comparison	Participants, n	Effect estimate	Certainty	Main reasons for downgrading
VO_2_ peak^a^	RVH-CR^b^ versus usual care	107	MD^c^ 2.50 mL/kg/min, 95% CI 0.88 to 4.12	Low	Single-study evidence, risk of bias, and imprecision due to small sample size
VO_2_ peak	RVH-CR versus CBCR^d^	251	MD 0.82 mL/kg/min, 95% CI 0.06 to 1.57	Moderate	Limited number of trials and some risk-of-bias concerns
6MWD^e^	RVH-CR versus usual care	296	MD 22.99 m, 95% CI 1.15 to 44.82	Low	Risk-of-bias, heterogeneity, and imprecision
6MWD	RVH-CR versus CBCR	304	MD −5.06 m, 95% CI −17.99 to 7.86	Moderate	Some risk-of-bias concerns and limited number of trials
MLHFQ^f^	RVH-CR versus usual care	98	SMD^g^ −0.57, 95% CI −0.98 to −0.17	Low	Single-study evidence, risk of bias, subjective outcome assessment, and imprecision
MLHFQ	RVH-CR versus CBCR	173	SMD −0.16, 95% CI −0.55 to 0.23	Moderate	Subjective outcome assessment and some risk-of-bias concerns
SF-36^h^	RVH-CR versus usual care	875	SMD 0.14, 95% CI −0.07 to 0.34	Low	Subjective outcome assessment and imprecision because the CI included the null effect
SF-36	RVH-CR versus CBCR	131	SMD 0.14, 95% CI −0.21 to 0.48	Low	Single-study evidence, risk of bias, subjective outcome assessment, and imprecision
Mortality and serious adverse events	All comparisons, narrative synthesis	1368	No intervention-related deaths or serious adverse events were reported	Low	Few events, short follow-up, and incomplete or inconsistent event reporting

a VO_2_ peak: peak oxygen uptake.

b RVH-CR: remote, virtual, or hybrid cardiac rehabilitation.

c MD: mean difference.

d CBCR: center-based cardiac rehabilitation.

e 6MWD: 6-minute walk distance.

f MLHFQ: Minnesota Living with Heart Failure Questionnaire.

g SMD: standardized mean difference.

h SF-36: 36-Item Short Form Health Survey.

## Discussion

### Principal Results

This review synthesized evidence from 8 RCTs, including 1368 patients with HFrEF or HFrEF-predominant populations. In comparator-specific analyses, mHealth-supported RVH-CR showed a statistically significant improvement in VO_2_ peak compared with CBCR; however, this finding was based on a small number of trials and should be considered preliminary. Compared with usual care, mHealth-supported RVH-CR improved 6MWD, whereas observed changes in VO_2_ peak and MLHFQ were based on single-trial estimates and should be interpreted cautiously. No significant differences were observed for 6MWD or MLHFQ in comparisons with CBCR, and comparator-specific analyses did not indicate a clear effect on SF-36 scores. Because the included trials were not designed as noninferiority studies, these results should not be taken as evidence of equivalence or noninferiority. Although selected functional and HRQoL outcomes improved, the certainty of evidence was low to moderate due to small sample sizes, heterogeneity, and reliance on self-reported HRQoL measures. Across the trials, no deaths or serious adverse events were directly attributed to structured RVH-CR supported by mHealth; however, the limited number of events and short follow-up prevent definitive conclusions regarding safety.

The clinical significance of the statistically significant findings should be interpreted cautiously. The improvement in 6MWD versus usual care was approximately 23 m. In patients with chronic HF, a previous study estimated the minimal clinically important difference for the 6-minute walk test to be 30.1 m [35]. Therefore, the observed effect may be clinically relevant for some patients, although interpretation should consider baseline functional status, HF phenotype, comorbidities, and follow-up duration. The VO_2_ peak difference versus CBCR was statistically significant but small in absolute magnitude (MD 0.82 mL/kg/min) and was based on a limited number of trials. Therefore, this finding should be viewed as preliminary and should not be interpreted as evidence of clinically meaningful superiority over CBCR.

mHealth-supported RVH-CR may provide an access-oriented delivery option for structured CR, particularly for patients with geographic, logistical, or resource constraints [36,37]. Remote monitoring, exercise prescription, and telecommunication support may help deliver individualized exercise programs and improve selected functional outcomes, although the evidence for HRQoL remains mixed and outcome-specific [38,39]. Clinicians should recognize that improvements versus usual care reflect both structured exercise and digital support. In this context, mHealth should be viewed as the enabling technology layer supporting CR delivery, whereas RVH-CR describes the care delivery models [16,17,40].

### Comparison With Prior Literature

Previous systematic reviews and meta-analyses have studied telerehabilitation or eHealth interventions in patients with HF and other cardiac conditions. Most of these studies combined different types of interventions and patient populations. Li et al [21] evaluated phase II mHealth-supported CR across a broad cardiac population, including myocardial infarction, angina, revascularization, and HF, with interventions combining exercise, education, and remote monitoring. Rampengan et al [41] synthesized 37 RCTs evaluating eHealth interventions, including automated telemonitoring, human-led communication, and structured education, focusing on outcomes such as HF-related hospitalizations, mortality, quality of life, and HF-related knowledge. Although the review demonstrated a reduction in HF-related admissions, it did not specifically assess the effects of structured, exercise-based RVH-CR on functional capacity or exercise tolerance. Similarly, Gao et al [42] and Cavalheiro et al [43] assessed home-based or telerehabilitation programs broadly across cardiovascular populations, reporting improvements in functional capacity and quality of life, but without isolating structured exercise interventions in patients with HF. In contrast, this review focused specifically on patients with HFrEF or HFrEF-predominant HF populations, evaluating structured, exercise-based RVH-CR supported by mHealth. By restricting inclusion to interventions with a clearly defined exercise prescription and a prespecified HFrEF-predominant population, this review isolates the effects of structured exercise delivered outside traditional center-based settings while allowing direct comparison with CBCR.

The present review focused on HFrEF or HFrEF-predominant populations, whereas evidence for mHealth-supported CR in HFpEF remains limited and is beginning to emerge. For example, Subramanian et al [44] recently evaluated a 6-month mHealth-based CR program in older adults with HFpEF and reported improvements in quality of life but no significant improvement in 6MWD or VO_2_ peak in the overall cohort.

### Strengths and Limitations

This review has several limitations that should be considered when interpreting the findings. First, the small number of included studies (n=8) and the relatively modest sample sizes in individual trials limited statistical power. These constraints also precluded subgroup analyses and meta-regression to explore sources of heterogeneity or potential effect modifiers. This limitation contributed to GRADE downgrading of evidence certainty for most outcomes. Second, several key outcome comparisons, including VO_2_ peak versus usual care and MLHFQ versus usual care, relied on single-trial estimates rather than pooled effects. This substantially limits the strength of inference. The statistically significant VO_2_ peak finding in the CBCR comparison was based on a small number of trials. Therefore, this result should be interpreted as preliminary and hypothesis-generating rather than as definitive evidence of superiority over CBCR. Third, HRQoL outcomes were mostly assessed using self-reported questionnaires in unblinded participants. This introduces the risk of performance and detection bias and was a principal reason for GRADE downgrading of HRQoL outcomes. Fourth, publication bias could not be formally evaluated using funnel plots or the Egger regression test because fewer than 10 studies were available for any single comparison. Fifth, although all included interventions involved structured exercise-based RVH-CR delivered through mHealth platforms, there was considerable heterogeneity in the specific technologies, monitoring strategies, feedback mechanisms, and rehabilitation protocols used across studies. Sixth, the findings apply to HFrEF or HFrEF-predominant HF populations and should not be generalized to HFpEF, for which mHealth-supported CR evidence is still emerging and requires dedicated synthesis.

Despite these limitations, this review has notable strengths that enhance its clinical and methodological value. Unlike prior systematic reviews that examined telerehabilitation broadly across heterogeneous cardiac populations or combined multiple intervention modalities, this review specifically focused on structured, exercise-based RVH-CR supported by mHealth in patients with HFrEF or HFrEF-predominant populations with HF. This allowed for a more targeted and clinically meaningful synthesis. All included interventions incorporated a clearly defined exercise prescription with patient-generated data capture or clinician-mediated feedback, isolating the effects of structured exercise from general digital health support. RoB was assessed using the Cochrane RoB 2 tool, and evidence certainty was evaluated using the GRADE framework, ensuring methodological transparency and reproducibility. Analyses were stratified by comparator type (CBCR vs usual care) to avoid interpretive confounding arising from pooling structurally distinct control conditions.

### Future Research Directions

Future research should prioritize larger, adequately powered RCTs with longer follow-up to assess the durability of mHealth-supported RVH-CR effects on exercise capacity, quality of life, cardiovascular mortality, and hospitalization in patients with HF. Standardization of mHealth platforms, exercise prescription protocols, and monitoring intensity across trials is essential to reduce heterogeneity and enable more definitive evidence synthesis. Studies should systematically report adherence rates, patient engagement, and intervention fidelity. Greater attention to underrepresented populations, including older adults, women, and individuals from low-income and middle-income settings, is needed to establish generalizability and support equitable implementation of mHealth-supported CR. Future systematic reviews should specifically evaluate mHealth-supported RVH-CR in HFpEF populations.

### Conclusions

The effects of structured RVH-CR supported by mHealth varied according to the comparator type in patients with HFrEF or HFrEF-predominant HF populations. Compared with usual care, mHealth-supported RVH-CR was associated with improved 6MWD, whereas single-trial estimates suggested improvements in VO_2_ peak and MLHFQ scores. Compared with CBCR, mHealth-supported RVH-CR showed a statistically significant greater improvement in VO_2_ peak in a limited number of trials, whereas no statistically significant differences were detected for 6MWD, MLHFQ, or SF-36. Because usual care and CBCR are clinically distinct comparators, these findings should not be interpreted as a single overall effect across all control conditions. No intervention-related deaths or serious adverse events were reported in the included trials, but its safety still needs to be studied. Equivalence or noninferiority to CBCR cannot be inferred from the available evidence. Future studies should evaluate long-term clinical outcomes and standardize intervention protocols.

## Supplementary material

10.2196/90422 Multimedia Appendix 1 Search strategy.

10.2196/90422 Multimedia Appendix 2 Characteristics of the included randomized controlled trials.

10.2196/90422 Multimedia Appendix 3 Risk of bias tool version 2 summary table.

10.2196/90422 Multimedia Appendix 4 GRADE (Grading of Recommendations Assessment, Development, and Evaluation) result table.

10.2196/90422 Checklist 1 PRISMA checklist.

## Abbreviations

6MWD: 6-minute walk distance
CBCR: center-based cardiac rehabilitation
CR: cardiac rehabilitation
GRADE: Grading of Recommendations Assessment, Development, and Evaluation
HF: heart failure
HFpEF: heart failure with preserved ejection fraction
HFrEF: heart failure with reduced ejection fraction
HRQoL: health-related quality of life
MD: mean difference
mHealth: mobile health
MLHFQ: Minnesota Living with Heart Failure Questionnaire
VO_2_ peak: peak oxygen uptake
PRISMA: Preferred Reporting Items for Systematic Reviews and Meta-Analyses
RCT: randomized controlled trial
RoB 2: risk of bias tool version 2
RVH-CR: remote, virtual, or hybrid cardiac rehabilitation
SF-36: 36-Item Short Form Health Survey
SMD: standardized mean difference
TELEREH-HF: Telerehabilitation in Heart Failure Patients
