# Supplementary material for: Effects of Remote, Virtual, or Hybrid Cardiac Rehabilitation Supported by mHealth in Patients With Heart Failure: Systematic Review and Meta-Analysis
Source: JMIR Mhealth Uhealth. 2026 Jul 21;14:e90422. doi: 10.2196/90422 (PMC13387639; doi:10.2196/90422)
Supplement: Multimedia Appendix 1 [file mhealth-v14-e90422-s001.docx]

PubMed

#1 ("heart failure" OR "cardiac failure" OR CHF OR HFrEF OR HFpEF)

#2 ("cardiac rehabilitation" OR "exercise training" OR "exercise rehabilitation" OR "exercise-based rehabilitation")

#3 (home-based OR "home based" OR HBCR OR telerehabilitation OR tele-rehabilitation OR telehealth OR telemedicine OR remote OR digital OR mHealth OR eHealth OR smartphone OR app OR wearable OR "text message" OR SMS)

#4 (random* OR trial OR "controlled trial")

Web of Science Core Collection

TS=(("heart failure" OR "cardiac failure" OR CHF OR HFrEF OR HFpEF)
AND ("cardiac rehabilitation" OR "exercise training" OR "exercise-based rehabilitation")
AND ("mobile health" OR mHealth OR eHealth OR "digital health" OR telehealth OR telemedicine OR telerehabilitation OR smartphone OR app OR wearable OR "remote monitoring" OR SMS OR "text message")
AND (home-based OR "home based" OR HBCR OR home)
AND (random* OR trial))

MEDLINE

1. exp Heart Failure/ OR "heart failure".ti,ab. OR "cardiac failure".ti,ab. OR "chronic heart failure".ti,ab. OR CHF.ti,ab. OR HFrEF.ti,ab. OR HFpEF.ti,ab.
2. exp Cardiac Rehabilitation/ OR "cardiac rehabilitation".ti,ab. OR "exercise rehabilitation".ti,ab. OR "exercise-based rehabilitation".ti,ab. OR rehabilitation.ti,ab.
3. exp Home Care Services/ OR "home care".ti,ab. OR "home-based cardiac rehabilitation".ti,ab. OR HBCR.ti,ab. OR "home rehabilitation".ti,ab. OR telerehabilitation.ti,ab. OR tele-rehabilitation.ti,ab. OR "telehealth".ti,ab. OR telemedicine.ti,ab.
4. exp Mobile Health/ OR "mobile health".ti,ab. OR mHealth.ti,ab. OR "digital health".ti,ab. OR eHealth.ti,ab. OR smartphone.ti,ab. OR app.ti,ab. OR "mobile phone".ti,ab. OR "wearable technology".ti,ab. OR "text message".ti,ab. OR SMS.ti,ab. OR "remote monitoring".ti,ab.
5. exp Randomized Controlled Trials/ OR "randomized controlled trial".ti,ab. OR random*.ti,ab. OR trial.ti,ab. OR "controlled clinical trial".ti,ab.
6. 1 AND 2 AND 3 AND 4 AND 5

Cochrane CENTRAL

#1 ("heart failure" OR "cardiac failure" OR CHF OR HFrEF OR HFpEF)

#2 ("cardiac rehabilitation" OR "exercise training" OR "exercise-based rehabilitation")

#3 ("mobile health" OR mHealth OR eHealth OR "digital health" OR telehealth OR telemedicine OR telerehabilitation OR smartphone OR app OR wearable OR "remote monitoring" OR SMS OR "text message")

#4 (home-based OR "home based" OR HBCR OR home)

CINAHL Complete

(MH "Heart Failure+") OR TI ("heart failure" OR "cardiac failure" OR "congestive heart failure" OR CHF) OR AB ("heart failure" OR "cardiac failure" OR "congestive heart failure" OR CHF)

AND

(MH "Cardiac Rehabilitation") OR TI ("cardiac rehabilitation" OR "cardiac rehab" OR CR) OR AB ("cardiac rehabilitation" OR "cardiac rehab" OR CR)

AND

(MH "Telehealth+") OR (MH "Mobile Applications") OR (MH "Smartphone") OR (MH "Text Messaging")

OR TI (mHealth OR "mobile health" OR telehealth OR telemedicine OR telerehabilitation OR "tele-rehabilitation" OR "mobile app*" OR smartphone* OR "text messag*" OR SMS OR wearable* OR "remote monitoring")

OR AB (mHealth OR "mobile health" OR telehealth OR telemedicine OR telerehabilitation OR "tele-rehabilitation" OR "mobile app*" OR smartphone* OR "text messag*" OR SMS OR wearable* OR "remote monitoring")

AND

(MH "Home Rehabilitation") OR TI ("home-based" OR homebased OR "home based" OR remote OR virtual OR community-based) OR AB ("home-based" OR homebased OR "home based" OR remote OR virtual OR community-based)
