# Supplementary material for: Effects of Remote, Virtual, or Hybrid Cardiac Rehabilitation Supported by mHealth in Patients With Heart Failure: Systematic Review and Meta-Analysis
Source: JMIR Mhealth Uhealth. 2026 Jul 21;14:e90422. doi: 10.2196/90422 (PMC13387639; doi:10.2196/90422)
Supplement: Multimedia Appendix 2 [file mhealth-v14-e90422-s002.docx]

|  | Country | Total  sample size  (intervention:  control), n | Mean  age (SD),  years | Male  participants,  n (%) or  n/N (%)* | Mean  LVEF (SD), % | Intervention  duration | CR session  frequency | Type of mHealth intervention | Control group | Primary and  secondary  outcomes | |
| --- | --- | --- | --- | --- | --- | --- | --- | --- | --- | --- | --- |
| **CBCR** | | | | | | | | | | |  |
| Hwang et al. (2017) | Australia | 53 (24:29) | 67 (12) | 40 (75%) | 35(17) | 12 weeks | Twice weekly | Participants were loaned a laptop, 3G mobile broadband, blood pressure monitor, fingertip pulse oximeter, and dumbbells/resistance bands; exercise was supervised by a physiotherapist via internet-based videoconferencing; education was delivered using electronic slides and audio. | Centre-based CR (CBCR), including education plus individualized aerobic and resistance exercise training. | Primary: 6MWT. Secondary: other functional measures, muscle strength, QoL, urinary incontinence, patient satisfaction, attendance, and adverse events. | |
| Schmidt et al. (2025) | Portugal | 120 (75:45) | 62.3 (10.8) | 76 (63%) | HFrEF: 103  HFpEF: 11  36.4(11.3) | 12 weeks | Twice weekly | 4 supervised sessions plus 20 home sessions; training data were recorded with a wearable smartwatch and uploaded to Polar Flow; lifestyle counselling/follow-up was delivered by telephone; home training was asynchronously monitored. | CBCR: 24 supervised exercise sessions including warm-up, resistance training (elastic bands; 2 sets of 10 exercises), and moderate- to high-intensity aerobic training. | Primary: change in VO2peak. Secondary: 6MWD, MLHFQ/HR-QoL, disease-related biomarkers, and physical fitness. | |
| Piotrowicz et al. (2010) | Poland | 152 randomized; 131 included in final analysis (75:56) | Overall: 58.1 (10.2); final analysis: HBCR 56.4 (10.9), CBCR 60.5 (8.8) | 117 (89%) in final analysis sample | HBCR 30.2(8.2)  CBCR 30.8(6.7) | 8 weeks | Three times weekly | EHO-3 device plus mobile phone; three precordial ECG leads were recorded and transmitted by phone to a monitoring centre; the training programme was preset with audiovisual prompts; the centre could adjust workload or stop training; telephone contact also provided psychological support. | Centre-based CR (SCR): interval cycle-ergometer training including 5-10 min warm-up, 10-30 min aerobic endurance training, and 5 min cool-down. | NYHA class, VO2peak, 6MWT distance, and SF-36/HR-QoL. | |
| **usual care** | | | | | | | | | | |  |
| Piotrowicz et al. (2021) | Poland | 850 randomized; QoL analysis included 768 (377:391) | HBCR 62.2 (10.9); UC 62.1 (10.2) | 684 (89%) in QoL analysis sample | HBCR 31(6.9)  UC 30.3(7) | 9 weeks (1-week in-hospital initial phase plus 8 weeks at home) | Five times weekly | Telecare (including psychological telesupport), individualized telerehabilitation, and CIED telemonitoring; devices included EHO-MINI tele-ECG training equipment, blood pressure monitor, weighing scale, mobile phone, and monitoring centre; training included aerobic endurance/Nordic walking, inspiratory muscle training, and light resistance/strengthening exercises. | Usual care/observation with lifestyle modification and self-management advice. | SF-36 QoL domains; psychological measures included BDI (QoL analysis). | |
| Nagatomi et al. (2022) | Japan | 30 (15:15) | 63.7 (10.1) | 16 (53%) | HFrEF: 14  42.2(17.4) | 3 months | Approximately weekly multidisciplinary communication; training frequency was individualized (aerobic exercise approximately 3-5 times/week, adjusted according to the protocol) | Fitbit Inspire HR monitoring; patient self-monitoring of symptoms, blood pressure, weight, steps, and training records; therapists communicated via app messages/telephone approximately weekly and adjusted training frequency/intensity based on symptoms and pulse data; dietitians provided individualized nutrition advice based on meal photographs. | Usual care, including pharmacological and non-pharmacological therapy. | Primary: change in 6MWD. Secondary: BNP, KCCQ, SPPB, Kihon Checklist, and related measures. | |
| Peng et al. (2018) | China | 98 (49:49) | 66.3 (10.5) | 58 (59%) | HBCR 34.03(6.64)  UC 34.07(6.66) | 8 weeks (2 months); follow-up to 6 months after discharge | Weeks 1-4: 3 times/week, 20 min/session; weeks 5-8: 5 times/week, 30 min/session | QQ/WeChat and webcam communication/supervision; endurance training (walking/jogging) was provided during the first 4 weeks, followed by resistance and muscle-strength training during weeks 5-8; physiotherapists monitored, assessed, and adjusted intensity; cardiac nurses provided weekly follow-up, and patients could interact and consult at any time. | Usual care, including discharge education and routine outpatient follow-up. | Primary: MLHFQ/QoL. Secondary: 6MWD, resting heart rate, HADS, LVEF, and NYHA class. | |
| Piotrowicz et al. (2014) | Poland | 111 randomized; 107 included in final analysis (75:32) | TG 54.4 (10.9); CG 62.1 (12.5) | 95 (89%) in final analysis sample | HBCR 30(8)  UC 34(6) | 8 weeks | Five times weekly | EHO-MINI, blood pressure monitor, weighing scale, and mobile phone; the device contained an individualized training workflow (exercise/rest/ECG recording time points); ECG was transmitted immediately after training to the monitoring centre for programme assessment; telephone contact also provided psychological support. | Usual care: advice on lifestyle modification and self-management; no supervised exercise training. | Primary: VO2peak. Secondary: CPET exercise duration, 6MWT, QoL/SF-36, safety, adherence, and acceptability. | |
| Lundgren et al. (2023) | Norway | 61 (31:30) | 67.6 (11.3) | Approximately 50 (82%; 18% female) | HFrEF: 44  HFpEF: 5  HBCR 36.4(12.1)  UC 32.4(11) | 3-month intervention plus 3-month post-intervention follow-up | Twice weekly, 24 sessions in total (completion could be extended in case of delays) | group exercise was supervised remotely by physiotherapists using online videoconferencing; tablet/network equipment supported two-way audio/video; all participants attended a 2-day Living with Heart Failure course. | Usual care; the control group received no additional intervention beyond standard care and was encouraged to exercise according to guidelines. | Feasibility: adherence, adverse events, patient-reported safety/confidence, and technical issues. Exploratory: GSE, VO2peak, 6MWT, and physical activity. | |

Values are presented as mean (SD) unless otherwise indicated. Randomized and final-analysis samples are both reported when available.

Abbreviations: 6MWD, 6-minute walking distance; 6MWT, 6-minute walk test; BDI, Beck Depression Inventory; BNP, B-type natriuretic peptide; CBCR, centre-based cardiac rehabilitation; CHF, chronic heart failure; CIED, cardiac implantable electronic device; CPET, cardiopulmonary exercise testing; CR, cardiac rehabilitation; ECG, electrocardiography; EQ-5D, EuroQol 5-Dimension; GSE, General Self-Efficacy Scale; HADS, Hospital Anxiety and Depression Scale; HR-QoL, health-related quality of life; ICT, information and communication technology; IQR, interquartile range; KCCQ, Kansas City Cardiomyopathy Questionnaire; LVEF, left ventricular ejection fraction; MLHFQ, Minnesota Living with Heart Failure Questionnaire; MVPA, moderate-to-vigorous physical activity; NYHA, New York Heart Association; QoL, quality of life; RCT, randomized controlled trial; SCR, supervised cardiac rehabilitation; SF-36, 36-item Short Form Health Survey; SPPB, Short Physical Performance Battery; UC, usual care; VO2peak, peak oxygen uptake; HFrEF, heart failure with reduced ejection fraction; HFpEF, heart failure with preserved ejection fraction
