# Supplementary material for: Effects of Remote, Virtual, or Hybrid Cardiac Rehabilitation Supported by mHealth in Patients With Heart Failure: Systematic Review and Meta-Analysis
Source: JMIR Mhealth Uhealth. 2026 Jul 21;14:e90422. doi: 10.2196/90422 (PMC13387639; doi:10.2196/90422)
Supplement: Multimedia Appendix 3 [file mhealth-v14-e90422-s003.docx]

| Study | D1 Randomization process | D2 Deviations from intended interventions | D3 Missing outcome data | D4 Measurement of the outcome | D5 Selection of the reported result | Overall judgement | Key rationale |
| --- | --- | --- | --- | --- | --- | --- | --- |
| Hwang 2017 | Low risk | Low risk | Low risk | Low risk | Low risk | Low risk | The study reported random allocation, allocation concealment, intention-to-treat analysis, and blinding of outcome assessors. The primary outcome was the 6-minute walk distance (6MWD), which was measured by independent assessors, and attrition at the 12- and 24-week follow-ups was limited. The trial was registered (ACTRN12613000390785). It should be noted that, for adverse events, the authors stated that only falls had been prespecified in the registered protocol; however, other non-prespecified adverse events were transparently reported in the article. This issue does not affect the judgement for the primary 6MWD outcome. |
| Lundgren 2023 | Low risk | Some concerns | Some concerns | Some concerns | Low risk | Some concerns | Randomization was stratified by age and LVEF and implemented by the NTNU Unit for Applied Clinical Research; the trial was registered at ClinicalTrials.gov. Blinding of the intervention was infeasible, and the control group received usual care only, without equivalent contact. CPET assessment was blinded, but the 6MWT/SPPB was conducted by non-blinded research personnel, and self-reported outcomes such as perceived safety and satisfaction could have been influenced by awareness of group allocation. The number of participants included in follow-up analyses decreased from 61 to approximately 53; although reasons were reported and ITT/mixed-model analyses were used, the sample size was small. |
| Nagatomi 2022 | Some concerns | Some concerns | Low risk | Some concerns | Low risk | Some concerns | This was a single-center, open-label RCT using stratified block randomization based on baseline 6MWD >=400 m, with data managed in REDCap. However, allocation concealment was not explicitly described, and participants in the HBCR group were significantly younger than those in the control group. All 30 participants completed the 3-month assessment, indicating a low risk from missing data. The primary outcome, 6MWD, is relatively objective; nevertheless, because the trial was open label and assessor blinding was not reported, concerns regarding measurement bias remain. The protocol was registered in UMIN. |
| Peng 2018 | Low risk | Some concerns | Some concerns | High risk | Some concerns | High risk | The study used a computer-generated random sequence and sequentially numbered opaque envelopes, and data collectors, data analysts, and outcome assessors were blinded. However, patients could not be blinded to the intervention. The primary outcome was the MLHFQ/QoL, a self-reported subjective outcome for which awareness of allocation could directly influence reporting. Of 98 randomized participants, 83 were included in the final analysis, which was based on complete cases rather than a clearly specified ITT approach. No trial registration or prepublished protocol information was identified in the article. |
| Piotrowicz 2010 | Some concerns | Some concerns | High risk | Some concerns | Some concerns | High risk | The study was reported as randomized; however, details on sequence generation and allocation concealment were insufficient. All 77 participants in the HTCR group completed rehabilitation, whereas 15 of 75 participants (20%) in the SCR group withdrew, mainly because of transport/cost constraints and difficulties with daily scheduling. The final analysis included 75 participants in the HTCR group and 56 in the SCR group, indicating substantial differential missingness and a complete-case analysis. The main exercise-capacity outcomes were relatively objective, but blinding of outcome assessors was not clearly described. No trial registration, protocol, or clearly prespecified primary outcome was identified. |
| Piotrowicz 2014 | Some concerns | Some concerns | Low risk | Low risk | Some concerns | Some concerns | The study used a random-number table with a 2:1 allocation ratio, but allocation concealment was not reported. The trial was not blinded; only CPET was performed by technicians unaware of group allocation. Baseline imbalances were present for age, LVEF, heart failure etiology, ICD use, and CPET exercise duration, although adjusted analyses were subsequently performed. Missing data were limited, and the primary outcome, VO2peak, was an objective measure assessed with blinded procedures. Because no trial registration or protocol was identified, the risk of selective reporting was judged as some concerns. |
| Piotrowicz 2021 | Some concerns | Some concerns | Some concerns | High risk | Some concerns | High risk | This article was a QoL subanalysis of the TELEREH-HF randomized clinical trial, a multicenter, open-label, parallel-group trial that had been registered. However, this article did not provide detailed information on random sequence generation or allocation concealment. Among 850 randomized participants, the final QoL analysis included 377 in the HCTR group and 391 in the UC group; 27 participants in the HCTR group did not receive HCTR, and additional participants did not complete questionnaires. The main outcomes were self-reported QoL measures, including SF-36 domains. Under open-label conditions, the risk of measurement bias was high. Because this was a subanalysis involving multiple QoL dimensions, selective reporting was judged as some concerns. |
| Schmidt 2025 | Low risk | Low risk | Some concerns | Low risk | Low risk | Some concerns | The study was a single-center, single-blind, non-inferiority RCT. Randomization was performed by an independent researcher using MinimPy minimization, and staff remained blinded except for personnel delivering the intervention. The protocol was registered and the study was reported according to CONSORT. The primary outcome, VO2peak, was an objective CPET measure. The main concern was that 12-week VO2peak data were available for 95 of 120 randomized participants (79%), with 34/45 in the center-based group and 61/75 in the home-based group. Because the proportion of missing outcome data exceeded the ideal level, D3 was judged as some concerns. |

**D1: Randomization process.** Hwang 2017, Lundgren 2023, Peng 2018, and Schmidt 2025 provided adequate reporting of the randomization methods or the personnel responsible for allocation. Specifically, Hwang explicitly reported allocation concealment, Peng used a computer-generated randomization sequence and opaque envelopes, and Schmidt used minimization performed by an independent researcher. Nagatomi 2022 described stratified block randomization and REDCap-based data management, but did not clearly report allocation concealment and showed a baseline age imbalance. Piotrowicz 2010 stated only that participants were randomly assigned, without details of sequence generation or allocation concealment; Piotrowicz 2014 used a random-number table but did not report allocation concealment and had multiple baseline imbalances; and Piotrowicz 2021 was a subanalysis of the main trial in which randomization procedures were not sufficiently detailed in the article. These studies were therefore judged as raising some concerns.

**D2: Deviations from intended interventions.** Given the nature of cardiac rehabilitation/telerehabilitation interventions, blinding of participants and personnel was difficult in all studies. Hwang 2017 and Schmidt 2025 used active controls or relatively comparable rehabilitation prescriptions and reported ITT or similar analyses; therefore, the risk in D2 was low. In Lundgren 2023, Nagatomi 2022, Peng 2018, and the Piotrowicz studies, control groups mostly received usual care or center-based rehabilitation, with incomplete comparability in contact frequency, psychological/nutritional support, and remote feedback. Some studies also did not fully implement ITT analyses; accordingly, most were judged as having some concerns.

**D3: Missing outcome data.** Follow-up data were relatively complete in Hwang 2017 and Nagatomi 2022, and the proportion of missing data was small in Piotrowicz 2014; thus, the risk was low. Lundgren 2023, Peng 2018, Piotrowicz 2021, and Schmidt 2025 had some missing data or included only complete cases. Although reasons for missing data were reported in some studies, small sample sizes or differential missingness could still compromise the stability of effect estimates. In Piotrowicz 2010, 20% of participants in the SCR group withdrew, and withdrawal was directly related to transport costs and the feasibility of attending center-based rehabilitation. The final analysis excluded a substantial number of randomized participants; therefore, D3 was judged as high risk.

**D4: Measurement of the outcome.** For objective exercise-capacity outcomes, such as CPET VO2peak and 6MWD, risk is generally low when assessor blinding or standardized measurement is adequate. Hwang 2017, Piotrowicz 2014, and Schmidt 2025 performed relatively well in this respect. Nagatomi 2022 did not report blinding of 6MWD assessors; in Lundgren 2023, CPET was blinded, but the 6MWT/SPPB was performed by unblinded personnel. These studies were therefore judged as having some concerns. In Peng 2018 and Piotrowicz 2021, the main evaluation focus was self-reported QoL outcomes. Because participants were aware of group allocation, subjective outcomes were susceptible to expectation effects and intervention-contact effects; therefore, D4 was judged as high risk.

**D5: Selection of the reported result.** Hwang 2017, Lundgren 2023, Nagatomi 2022, and Schmidt 2025 reported trial registration or published protocols, and their primary outcomes were clearly specified; therefore, the risk of selective reporting was low. Peng 2018, Piotrowicz 2010, and Piotrowicz 2014 did not provide sufficient information on trial registration or prepublished protocols in the article. Piotrowicz 2021 was derived from a registered main trial, but it was a QoL subanalysis involving multiple SF-36 domains, and it was not sufficiently clear whether all analyses had been prespecified. Accordingly, D5 was judged as some concerns.
