# Supplementary material for: Effects of Remote, Virtual, or Hybrid Cardiac Rehabilitation Supported by mHealth in Patients With Heart Failure: Systematic Review and Meta-Analysis
Source: JMIR Mhealth Uhealth. 2026 Jul 21;14:e90422. doi: 10.2196/90422 (PMC13387639; doi:10.2196/90422)
Supplement: Multimedia Appendix 4 [file mhealth-v14-e90422-s004.docx]

| Outcome | No. of Studies / Participants | Risk of Bias | Inconsistency | Indirectness | Imprecision | Publication Bias | Overall Certainty | Notes / Downgrade Reasons |
| --- | --- | --- | --- | --- | --- | --- | --- | --- |
| VO₂ peak vs Usual Care | 1 study / 107 participants | Serious – single trial, high risk of bias | Not applicable | No serious | Serious – small sample, wide CI | Undetected | Low | Single study; subjective outcome; small sample size |
| VO₂ peak vs CBCR | 2 studies / 251 participants | Some concerns – open-label, unclear allocation | Not serious | No serious | Not serious | Undetected | Moderate | Multiple studies; objective outcomes; minor risk of bias |
| 6MWD vs Usual Care | 4 studies / 296 participants | Some concerns – high attrition in some studies | Serious – high heterogeneity (I² = 67.72%) | No serious | Some concerns – CI wide | Undetected | Low | Heterogeneity across trials; sample size variation |
| 6MWD vs CBCR | 3 studies / 304 participants | Some concerns – open-label, incomplete outcome data | Not serious | No serious | Not serious | Undetected | Moderate | Low heterogeneity; objective outcome |
| MLHFQ vs Usual Care | 1 study / 98 participants | Serious – single study, high risk of bias | Not applicable | No serious | Serious – small sample, wide CI | Undetected | Low | Single study; subjective outcome; wide CI |
| MLHFQ vs CBCR | 2 studies / 173 participants | Some concerns – open-label, subjective outcome | Not serious | No serious | Not serious | Undetected | Moderate | Multiple studies; subjective outcome; minor risk of bias |
| SF-36 vs Usual Care | 2 studies / 875 participants | Some concerns – open-label, subjective outcome | Not serious | No serious | Serious – CI includes null effect | Undetected | Low | effect estimate imprecision |
| SF-36 vs CBCR | 1 study / 131 participants | Serious – single study, high risk of bias | Not applicable | No serious | Serious – small sample, CI includes null | Undetected | Low | Single study; small sample; subjective outcome |
| Mortality / Serious Adverse Events | 8 studies / 1,368 participants | Some concerns – short follow-up, incomplete event reporting | Not serious | No serious | Serious – few events | Undetected | Low | Few events; short-term follow-up; incomplete reporting |

**Legend / Downgrade Justification:**

- **Risk of Bias:** Open-label design, high attrition, reliance on self-reported outcomes.
- **Inconsistency:** High heterogeneity in effect estimates across trials.
- **Indirectness:** Population, interventions, and outcomes directly match research question.
- **Imprecision:** Small sample sizes or wide confidence intervals.
- **Publication Bias:** Not detected due to limited number of studies per outcome (<10).
